# Prognostic Factor Analysis and Model Construction of Triple-Negative Metaplastic Breast Carcinoma After Surgery

**DOI:** 10.3389/fonc.2022.924342

**Published:** 2022-06-23

**Authors:** Keying Zhu, Yuyuan Chen, Rong Guo, Lanyi Dai, Jiankui Wang, Yiyin Tang, Shaoqiang Zhou, Dedian Chen, Sheng Huang

**Affiliations:** The 2nd Department of Breast Surgery, Breast Cancer Center of the Third Affiliated Hospital of Kunming Medical University, Yunnan Cancer Hospital, Kunming, China

**Keywords:** metaplastic breast cancer, overall survival, nomogram, prognostic model, surgery

## Abstract

**Objective:**

The study aimed to analyze the prognostic factors of patients with triple-negative (TN) metaplastic breast carcinoma (MpBC) after surgery and to construct a nomogram for forecasting the 3-, 5-, and 8-year overall survival (OS).

**Methods:**

A total of 998 patients extracted from the Surveillance, Epidemiology, and End Results (SEER) database were assigned to either the training or validation group at random in a ratio of 7:3. The clinical characteristics of patients in the training and validation sets were compared, and multivariate Cox regression analysis was used to identify the independent risk variables for the OS of patients with TN MpBC after surgery. These selected parameters were estimated through the Kaplan–Meier (KM) curves using the log-rank test. The nomogram for predicting the OS was constructed and validated by performing the concordance index (C-index), receiver operating characteristics (ROC) curves with area under the receiver operating characteristic curves (AUC), calibration curves, and decision curve analyses (DCAs). Patients were then stratified as high-risk and low-risk, and KM curves were performed.

**Results:**

Multivariate Cox regression analysis indicated that factors including age, marital status, clinical stage at diagnosis, chemotherapy, and regional node status were independent predictors of prognosis in patients with MpBC after surgery. Separate KM curves for the screened variables revealed the same statistical results as with Cox regression analysis. A prediction model was created and virtualized *via* nomogram based on these findings. For the training and validation cohorts, the C-index of the nomogram was 0.730 and 0.719, respectively. The AUC values of the 3-, 5-, and 8-year OS were 0.758, 0.757, and 0.785 in the training group, and 0.736, 0.735, and 0.736 for 3, 5, and 8 years in the validation group, respectively. The difference in the OS between the real observation and the forecast was quite constant according to the calibration curves. The generated clinical applicability of the nomogram was further demonstrated by the DCA analysis. In all the training and validation sets, the KM curves for the different risk subgroups revealed substantial differences in survival probabilities (*P <*0.001).

**Conclusion:**

The study showed a nomogram that was built from a parametric survival model based on the SEER database, which can be used to make an accurate prediction of the prognosis of patients with TN MpBC after surgery.

## Introduction

Breast cancer (BC) is the leading cause of death in women, especially in women aged 20–59 years (1). Metaplastic breast cancer (MpBC) is a rare BC histological subtype with strong heterogeneity, accounting for approximately 0.3 to 1% of all kinds of BC (2). While MpBC is infrequent in terms of population incidence, it usually leads to an impoverished prognosis, consequently contributing to a relatively high mortality rate (3). “Metaplastic cancer” first appeared in 1973 by Huvos et al. (4). As defined by the World Health Organization (WHO), MpBC combines the presence of at least two histological cell types with metaplastic changes to squamous and/or mesenchymal elements (3). Additionally, they are highly heterogeneous and vary from chondroid, osseous, spindle, squamous, to rhabdomyoid elements (5). According to the biological behavior and histopathological characteristics of MpBC, malignant degrees can be divided into high and low (6). Most MpBCs are triple negative (TN) in phenotype, lack expression of estrogen receptor (ER), progesterone receptor (PR), and human epidermal growth factor receptor 2 (HER2) (7).

Generally, triple-negative breast cancer (TNBC) has always been considered a type with more aggressive behavior, a greater chance of recurrence, and a worse prognosis (8). Triple-negative metaplastic breast cancer (TN MpBC) is more resistant than other TNBCs to conventional chemotherapy and carries a worse prognosis with a doubled risk of local recurrence and distant metastasis (34% vs. 15.5%) (9). A study of 59,519 patients by Giovanni et al. clearly showed us that MpBC was associated with worse OS compared to TNBC with a significant 40% increased risk of death (10). TN MpBC differs from other typical and common BCs in the pathological and clinical aspects. However, the prognosis and predictive factors for it remain largely unknown. Therefore, an accurate prediction model of TN MpBC is urgently needed.

Nomograms have been extensively endorsed and supported as a forecasting method in a considerable range of diseases in oncology recently, such as endometrial sarcoma (11), BC (12), lung cancer (13), gallbladder cancer (14), prostate cancer (15), kidney carcinoma (16), etc. They have shown a high discriminative ability to predict survival in validation and meet the requirements for an integrated model. By creating a concise and direct evaluation graph, the nomogram can assist clinicians and patients in making the most optimal and informed decisions regarding treatment. The Surveillance, Epidemiology, and End Results (SEER) database is one of the most comprehensive and complete large-scale tumor registries in North America, gathering a vast quantity of evidence-based and medicine-related data with an approximate coverage of one-third of the U.S. population (17). In this study, we used the SEER database to create a nomogram that could predict the prognosis of patients with TN MpBC.

## Methods

### Patient Selection Criteria

Patients diagnosed with TN MpBC between 2010 and 2015 were initially identified using SEER*Stat (version 8.3.9) from the SEER database, which was all derived from 18 population-based cancer registries.

Inclusion criteria for patients with breast cancer were as follows: histology ICD-O-3 (8,575), triple-negative breast subtype, and surgery performed. Patients with N-adjusted, unknown adjusted AJCC 6th stage, unknown race recode, unknown tumor size, or positive regional nodes were excluded from this study.

### Cohort Definition and Variable Declaration

Eligible patients in the SEER database were randomly divided into training and validation cohorts with a 7:3 ratio by the “caret” package in R version 4.1.1. The training group was used to create the prediction model, and the validation cohort was performed to confirm the accuracy and applicability of the model. Thirteen variables were recorded to describe the characteristics of patients: age, sex, marital status at diagnosis, ethnicity, tumor size, clinical stage, surgery type, chemotherapy, radiotherapy, laterality of tumor, primary sites, historic stage, and positive regional nodes. The age of these patients was reclassified at 63 years old. Surgical types include breast-conserving surgery (BCS) and mastectomy. The tumor clinical stage was ranked according to the American Joint Committee on Cancer (AJCC) 6th Edition breast cancer staging criteria. The tumor size was divided into three categories: <20, 20–50, and >50 mm. Tumors were classified as primary locations in the central section of the breast, the lower-inner/lower-outer/upper-inner/upper-outer quadrant of the breast, and others. Moreover, the historic stage of the tumor was classified as localized, regional, and distant separately. As for regional nodes, negative or positive status was confirmed.

### Development of the Prognosis Model

Statistical analyses were conducted using the R software (4.1.2) and the SPSS 21.0. Significant factors were identified using Cox univariate analysis, and variables with *P <*0.05 were extracted into the multivariate Cox proportional hazards regression models. For each condition, the hazard ratios (HRs) and corresponding 95% confidence intervals (CIs) were calculated. On the basis of the results from prior multivariate analysis, the preferred independent risk variates were included in the nomogram to estimate the probability of the 3-, 5-, and 8-year MpBC OS rates following comprehensive therapy. The “rms” package was used to plot the nomogram. Selected patients were stratified as high and low risk, and the Kaplan–Meier (KM) curves with the log-rank test using the “survival” package of R software were performed to assess the significance of the overall survival (OS) difference between the low-risk and high-risk groups. OS was defined as the time gap between the date of diagnosis and the day of death from any cause or the final follow-up.

### Validation of Nomogram

The nomogram was then validated using various approaches. The concordance index (C-index) was generated to measure the predictive accuracy and discrimination capabilities. The receiver operating characteristic (ROC) curves were depicted, and the area under the receiver operating characteristic curves (AUC) was also created to examine the predictive accuracy. Commonly, C-index and AUC values greater than 0.7 indicate legitimate estimation. To test the association between the expected probabilities and the observed outcome frequencies, calibration curves were adapted. Decision curve analyses (DCAs) were performed to evaluate the clinical applicability and benefit of the nomogram.

## Results

### Patient Characteristics

The data on 998 patients with TN MpBC were taken from the SEER database and randomized into training and validation groups in a 7:3 ratio, according to the screening criteria. **Table 1** summarizes the demographic and clinical baseline features of these individuals. Almost all the included patients were women (99.6%). The median age of patients included in this cohort was 63 (inter-quartile range [IQR]:53–74). Most patients were white (77.1%), with localized tumor invasion (74.3%) or T stage II (62.9%). Moreover, 51.1% of patients were married, while 43.7% were not, and the rest of the information remained unknown. Additionally, the entire population had a relatively low risk of regional node metastasis (73.7%). Approximately 50.6 and 49.4% of the tumors were lateralized to the left and right, respectively, and most were located in the upper-outer quadrant of the breast (36.9%). In terms of therapy, mastectomy was performed in 57.9% of the patients, and the rest underwent BCS. Chemotherapy (65.9%) and radiotherapy (46.1%) were received by most of the included patients with TN MpBC (**Table 1**). There is a significant statistical difference in the age between the training and validation groups (*P <*0.05), whereas there is no significant difference in the distribution of the other described variables between the training and validation groups (**Table 1**).

**Table 1:** Characteristics of TN MpBC patients after surgery.

Variables	Total cohort (N = 998)		Training cohort (N = 700)		Validation cohort (N = 298)		*P-*value
	n	%	n	%	n	%	
Age (yrs.)							
<63	475	47.6	351	50.1	124	41.6	0.016*
≥63	523	52.4	349	49.9	174	58.4	
Race							
Black	163	16.3	111	15.9	52	17.4	0.651
Other	66	6.6	49	7	17	5.7	
White	769	77.1	540	77.1	229	76.8	
Sex							
Female	995	99.7	697	99.6	298	100	0.559
Male	3	0.3	3	0.4	0	0	
Marital status							
Unmarried	436	43.7	306	43.7	130	43.6	0.741
Married	514	51.5	358	51.1	156	52.3	
Unknown	48	4.8	36	5.1	12	4	
Clinical stage							
I	232	23.2	156	22.3	76	25.5	0.330
II	615	61.6	440	62.9	175	58.7	
III	122	12.2	87	12.4	35	11.7	
IV	29	2.9	17	2.4	12	4	
T							
T1	251	25.2	170	24.3	81	27.2	0.330
T2	499	50.0	354	50.6	145	48.7	
T3	160	16.0	119	17	41	13.8	
T4	88	8.8	57	8.1	31	10.4	
N							
N0	803	80.5	563	80.4	240	80.5	0.962
N1	133	13.3	92	13.1	41	13.8	
N2	35	3.5	25	3.6	10	3.4	
N3	27	2.7	20	2.9	7	2.3	
M							
M0	969	97.1	683	97.6	286	96	0.242
M1	29	2.9	17	2.4	12	4	
Tumor size (mm)							
≤20	254	25.5	172	24.6	82	27.5	0.607
20–50	523	52.4	370	52.9	153	51.3	
>50	221	22.1	158	22.6	63	21.1	
Primary site							
Central portion of breast/Nipple	51	5.1	35	5	16	5.4	0.619
Lower-inner	66	6.6	42	6	24	8.1	
Lower-outer	91	9.1	59	8.4	32	10.7	
Upper-inner	110	11.0	81	11.6	29	9.7	
Upper-outer	362	36.3	258	36.9	104	34.9	
Other	318	31.9	225	32.1	93	31.2	
Laterality							
Left	491	49.2	354	50.6	137	46	0.207
Right	507	50.8	346	49.4	161	54	
Surgery							
BCS	406	40.7	295	42.1	111	37.2	0.171
Mastectomy	592	59.3	405	57.9	187	62.8	
Radiotherapy							
No	554	55.5	377	53.9	177	59.4	0.123
Yes	444	44.5	323	46.1	121	40.6	
Chemotherapy							
No/unknown	355	35.6	239	34.1	116	38.9	0.170
Yes	643	64.4	461	65.9	182	61.1	
Historic stage							
Localized	736	73.7	520	74.3	216	72.5	0.757
Regional	215	21.5	149	21.3	66	22.1	
Distant	47	4.7	31	4.4	16	5.4	
Lymph nodes							
Negative	734	73.5	516	73.7	218	73.2	0.982
Positive	169	16.9	118	16.9	51	17.1	
No examined	95	9.5	66	9.4	29	9.7	

BCS, Breast-Conserving Surgery. *P<0.05, there is a the difference was statistically significant.

### Prognostic Variables Screening

**Figure 1:**
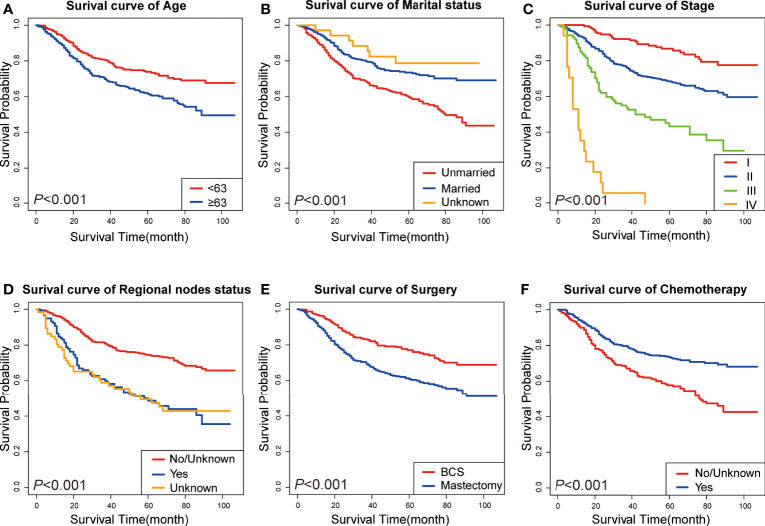
KM curves of prognostic factors in TN MpBC patients. **(A)** age; **(B)** marital status; **(C)** tumor stage; **(D)** regional nodes status; **(E)** surgery type; and **(F)** chemotherapy.

A Cox univariate survival analysis was performed for each variable in the training set. As demonstrated in **Table 2**, the Cox univariate regression results differentiated nine variables (age, marital status, clinical stage, tumor size, primary site, historic stage, surgery type, chemotherapy, and regional node status) that were substantially linked with TN MpBC OS (*P <*0.05). It is worth mentioning that tumor size, to some extent, corresponds to the T stage of clinical stage categorization. Thus, to avoid repetition, only TNM stage classification was included in our multivariate analysis rather than tumor size. These variables were then contained in the multi-factor Cox regression model. Based on the multivariate analysis, we ultimately ascertained that age ≥63 years (*P =* 0.094), unmarried status (*P =* 0.02), higher stage (*P <*0.01), positive regional nodes (*P =* 0.066), no chemotherapy (*P <*0.01), and mastectomy-received (*P =* 0.04) were independent risk variables in the poor prognosis of patients with TN MpBC (**Table 2**). KM curves drawn for the above six factors separately indicated the same results. As shown, patients younger than 63 years or who are married are more likely to survive longer than those older or unmarried (age [*P* <0.001], **Figure 1A**; marital status [*P* <0.001], **Figure 1B**). Additionally, survival rates decline with higher clinical stage (*P <*0.001) (**Figure 1C**) and positive regional lymph nodes (*P <*0.001) (**Figure 1D**). Different therapy types have different effects. Patients with MpBC who received BCS and chemotherapy tended to have a higher survival probability (surgery type [*P* <0.001], **Figure 1E**; and chemotherapy [*P* <0. 001], **Figure 1F**). These findings corroborate the statistical findings stated above. In summary, age, marital status, clinical stage, regional nodes, chemotherapy, and surgery type were significant factors that were associated with OS.

**Table 2:** Univariate and multifactorial Cox analysis of risk factors in TN MpBC patients after surgery.

Characteristics		Univariate analysis		Multivariate analysis		
		HR (95% CI)	*P*-value	HR (95% CI)	*P-*value	
Age (yrs.)				
<63		Reference		Reference		
≥63		1.644 (1.268–2.131)	<0.001^*^	1.283 (0.959–1.717)	0.094	
Race				
Black		Reference				
Other		0.580 (0.306–1. 098)	0.094			
White		0.808 (0.581–1.123)	0.205			
Sex				
Female		Reference				
Male		0.050 (0.000–882.829)	0.547			
Marital status				
Unmarried		Reference		Reference		
Married		0.547 (0.421–0.711)	<0.001^*^	0.625 (0.473–0.825)	0.001^*^	
Unknown		0.377 (0.177–0.807)	0.012^*^	0.520 (0.240–1.124)	0.096	
Clinical stage				
I		Reference		Reference		
II		2.336 (1.538–3.548)	<0.001^*^	2.968 (1.285–6.858)	0.011^*^	
III		5.256 (3.273–8.441)	<0.001^*^	4.497 (1.597–12.663)	0.004^*^	
IV		29.673 (15.809–55.694)	<0.001^*^	40.977 (11.044–152.045)	<0.001^*^	
T				
T1		Reference				
T2		1.808 (1.210–2.703)	0.004^*^			
T3		4.206 (2.734–6.472)	<0.001^*^			
T4		6.625 (4.149–10.579)	<0.001^*^			
N				
N0		Reference				
N1		1.674 (1.185–2.364)	0.003^*^			
N2		2.637 (1.554–4.476)	<0.001^*^			
N3		4.254 (2.542–7.119)	<0.001^*^			
M				
M0		Reference				
M1		12.654 (7.582–21.120)	<0.001^*^			
Tumor size (mm)				
≤20		Reference		Reference		
20–50		1.768 (1.205–2.594)	0.004^*^	0.653 (0.311–1.372)	0.261	
>50		4.284 (2.885–6.363)	<0.001^*^	1.196 (0.565–2.528)	0.640	
Primary site				
Central portion of breast/Nipple		Reference		Reference	
Lower-inner		0.766 (0.325–1.804)	0.542	1.268 (0.531–3.031)	0.593	
Lower-outer		1.373 (0.669–2.816)	0.388	1.737 (0.838–3.600)	0.138	
Upper-inner		0.861 (0.415–1.785)	0.687	1.749 (0.823–3.717)	0.146	
Upper-outer		1.029 (0.547–1.933)	0.93	1.594 (0.839–3.030)	0.155	
other		1.554 (0.832–2.901)	0.167	1.880 (0.999–3.539)	0.050^*^	
Surgery				
BCS		Reference		Reference	
Mastectomy		1.847 (1.404–2.431)	<0.001^*^	1.532 (1.145–2.051)	0.004^*^	
Radiotherapy				
No		Reference			
Yes		0.824 (0.638–1.065)	0.139		
Chemotherapy				
No/unknown		Reference		Reference	
Yes		0.531 (0.412–0.685)	<0.001^*^	0.582 (0.436–0.778)	<0.001^*^	
Historic stage				
Localized		Reference		Reference	
Regional		1.931 (1.448–2.574)	<0.001^*^	0.834 (0.485–1.435)	0.513	
Distant		6.540 (4.290–9.972)	<0.001^*^	0.761 (0.309–1.873)	0.553	
Lymph nodes				
negative		Reference		Reference	
positive		2.404 (1.785–3.238)	<0.001^*^	1.432 (0.890–2.304)	0.138	
no examined		2.454 (1.694–3.555)	<0.001^*^	1.586 (1.044–2.411)	0.031^*^	

BCS, Breast-Conserving Surgery. *P<0.05, there is a the difference was statistically significant.

### Nomogram Construction and Validation

**Figure 2:**
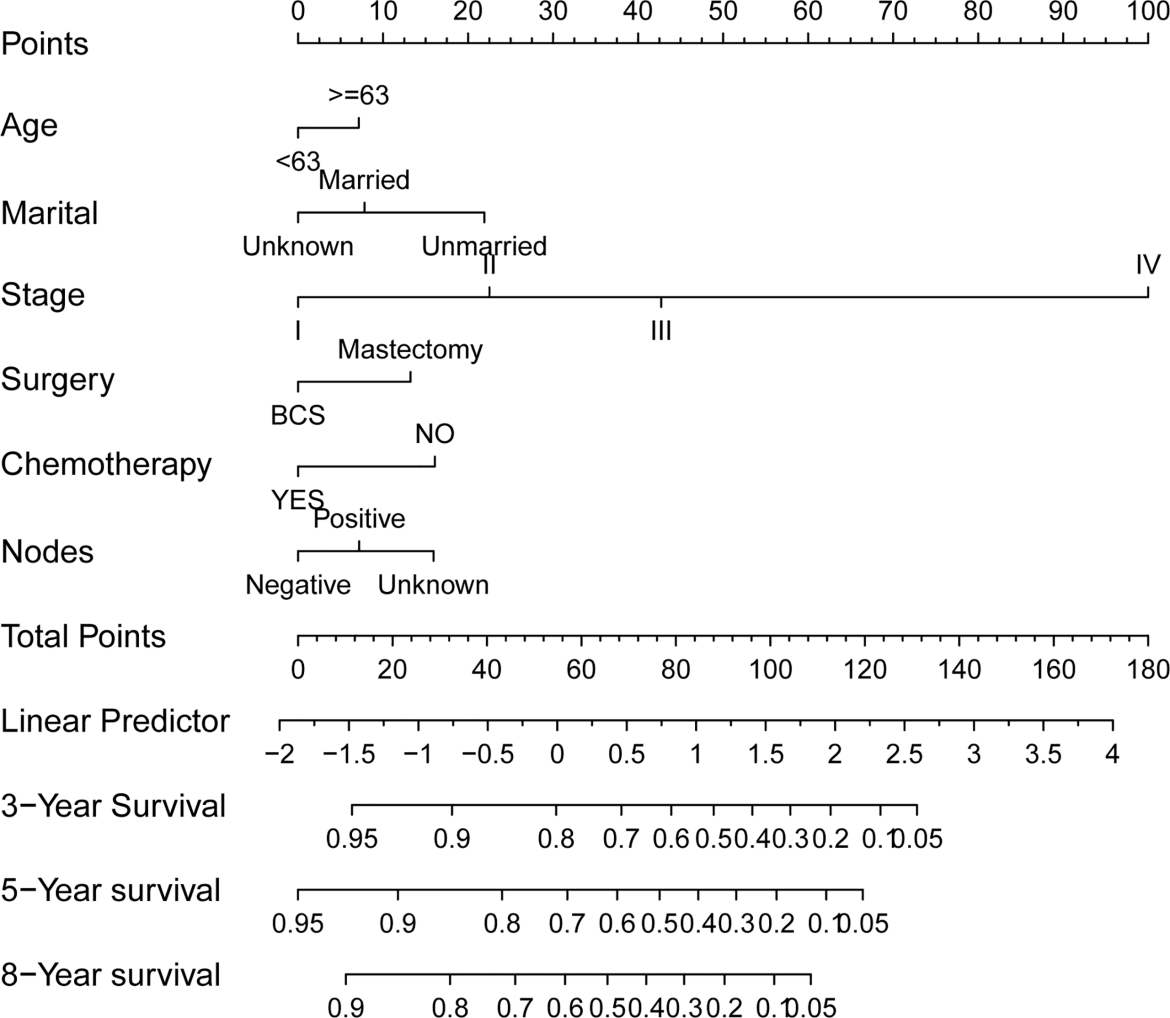
Prognostic nomograms of 3-, 5-, and 8-year OS in TN MpBC patients.

The preceding screened six factors were used to create a nomogram of OS prognosis in TN MpBC (**Figure 2**), and all the predictors were integrated to predict the 3- and 5-year survival of patients with MpBC.

**Figure 3:**
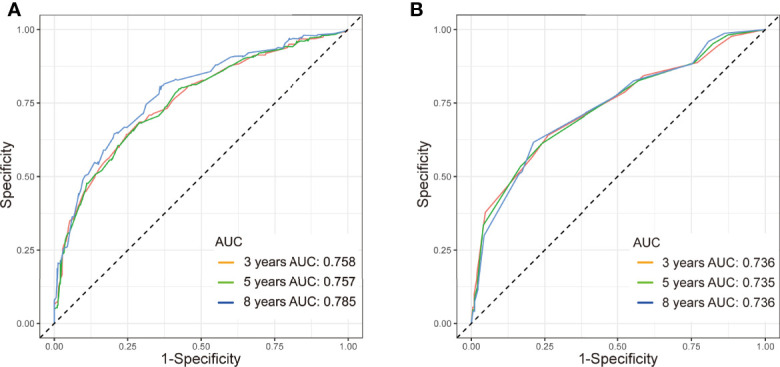
ROC curve with AUC for OS in TN MpBC patients. **(A)** 3-, 5-, and 8-year OS rate in the training set, **(B)** 3-, 5-, and 8-year OS rate in the validation set.

**Figure 4:**
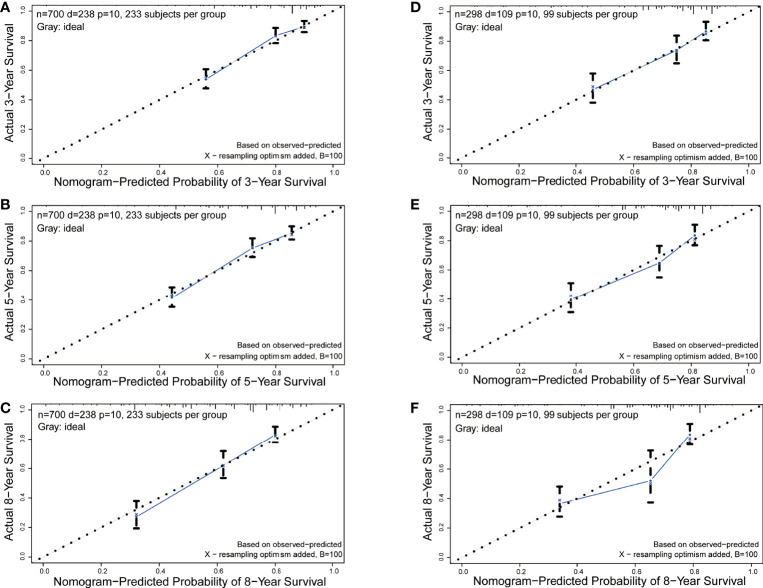
Calibration curves of the nomogram for 3-, 5-, and 8-year OS prediction. **(A-C)** 3-, 5-, and 8-year OS rate in the training set; **(D-F)** 3-, 5-, and 8-year OS rate in the validation set.

**Figure 5:**
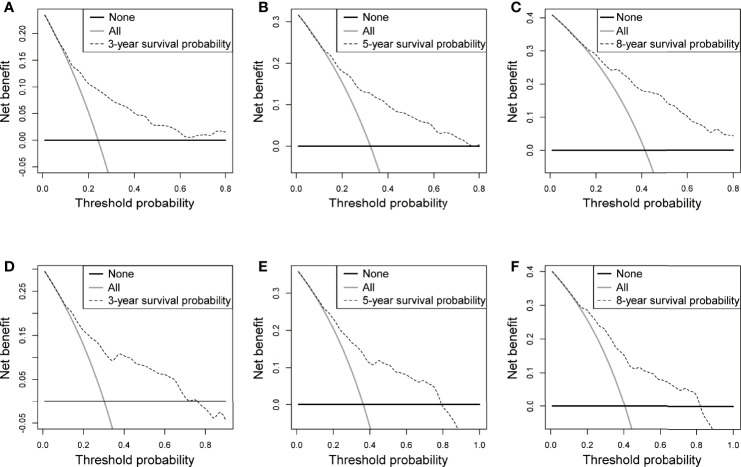
DCA in the training and validation sets. **(A-C)** 3-, 5-, and 8-year OS rate in the training set, **(D-F)** 3-, 5-, and 8-year OS rate in the validation set.

The C-index in the training and validation groups were 0.730 (95% CI: 0.713–0.747) and 0.719 (95% CI: 0.693–0.745), respectively, and this exhibited favorable prognostic accuracy and clinical practicality when we verified the discrimination of the nomogram. The above outcomes correspond to the ROC curves and the AUC value (**Figure 3**). The AUC value of 3-, 5-, and 10-year OS is higher than 0.70 and shows that the constructed nomogram has good predictive efficiency for OS. The AUC value of 3-year OS was 0.758 in the training cohort (*P <*0.001) (**Figure 3A**) and 0.736 in the validation cohort (*P <*0.001) (**Figure 3B**). For the 5-year OS, the AUC values in the training and validation groups were 0.757 (*P <*0.001) (**Figure 3A**) and 0.735 (*P <*0.001) (**Figure 3B**), respectively. The AUC value of the 8-year OS was 0.785 (*P <*0.001) (**Figure 3A**) and 0.736 (*P <*0.001) (**Figure 3B**), respectively. In both the training and validation cohorts, the calibration curves illustrated a high level of consistency between the actual observed results and the nomogram predictions of 3-, 5-, and 8-year OS (**Figures 4A–F**). Furthermore, DCA curves were plotted for the 3-, 5-, and 8-year survival of the training and validation sets. In addition, among virtually all of the threshold probabilities at different time periods, the DCA curves exhibited excellent net benefits in the predictive model, indicating an agreeable prospective clinical effect of the predictive model (**Figures 5A–F**).

### Risk Assessment

**Figure 6:**
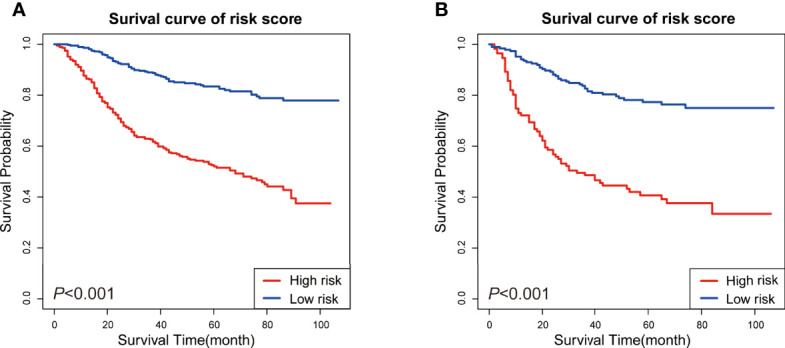
KM survival analysis of patients in different risk subgroups. **(A)** KM survival analysis in the training set, **(B)** KM survival analysis in the validation set.

According to former analysis and newly-built nomogram, we have performed a postoperative risk stratification, dividing patients into high- and low-risk groups. In both the training (*P <*0.001) and validation sets (*P <*0.001), the KM curves of the different risk subgroups indicated great survival probability distinction (**Figures 6A,B**). The high-risk group showed distinctly worse survival conditions than the low-risk group. These findings reveal that the risk classification system has a strong predictive value for the prognosis of patients with MpBC, further strengthening the potential applicability of this prognostic model.

## Discussion

MpBC is a rare, yet deadly form of BC that consists of epithelial and mesenchymal histological components (18). The WHO classified MpBC into the epithelial and epithelial–mesenchymal mixed types (19). According to its biobehavioral and histopathological features, MpBC can be subclassified into a high and low grade (3). Low-grade MpBC usually contains adenosquamous carcinoma and fibromatosis-like MpBC, while squamous cell carcinoma, spindle cell carcinomas, and heterologous mesenchymal differentiation comprise the highly malignant MpBC (20). For the MpBC pathogenesis, molecular alteration and genetic changes are usually taken into consideration. Some of these variations can be focused on as therapeutic targets preclinically and clinically (21). According to previous studies, MpBC typically has molecular variations in epithelial to mesenchymal transition (EMT) (22) and phosphoinositide 3-kinases (PI3K) signaling (23). Research has shown that the aggressiveness and poor clinical outcomes of MpBC can be explained by EMT characteristics along with PI3K pathway hyperactivation (24). Reis-Filho found that EGFR gene amplification, which is exhibited in nearly 34% of MpBC cases (25) and EGFR tyrosine kinase inhibitors, can be employed as therapeutics against MpBC. Other genetic differences also exist, such as nitric oxide signaling, Wnt/-catenin signaling, altered immunological response, and cell cycle dysregulation (21).

Since more than 90% of MpBC is negative for ER or PR and HER2 (26), and considering its rarity, research on clinical pathological features and the prognostic factors of patients with MpBC has been limited (3). Yet, no practical clinical diagnosis-treatment-prognosis guideline or consensus has been globally acknowledged, which has boosted enthusiasm for MpBC research recently. It is well known that all that TNBC has a poor prognosis among all molecular types of breast cancer (17). Existing studies have shown that the prognosis of MpBC is even worse than that of TNBC (27). Zhang et al. checked 30,000 patients with BC, and they discovered that the 5-year disease-free survival (DFS) rate and OS rate of the patients with MpBC were 67.9 and 78.7%, which is much lower than the 86.0 and 90.6% of healthy patients with TNBC, implying that the prognosis of MpBC was poorer than TNBC (28). This was consistent with the results from Pakha et al., where they found that the 5-year OS rate of MpBC was only 64%, which was significantly lower than that of IDC and TNBC (29). The long-term prognosis of patients is greatly influenced by MpBC immunohistological subgroups, with TN MpBC having the worst prognosis of all (30). As a result, it is of critical importance to investigate the factors that impact the prognosis of TN MpBC to identify patients at high risk.

In this study, 50.6 and 49.4% of the tumors were lateralized to the left and right, respectively, and most were located in the upper-outer quadrant of the breast (36.9%). This implies that no matter whether on the left or right breast, the upper-outer quadrant is the site where MpBC is most likely to occur. Besides, we found that in patients who included in this cohort, the median age was 63. In the training group, the age distribution was even for half who were older than 63 years old and the other half were younger. However, in the validation group, more patients were ≤63. There is a significant statistical difference in the age distribution. This indicates that age does not exert an influence on to the predictive efficiency of the predictive model. In our study, we confirmed that age, marital status, clinical stage, regional lymph nodes, chemotherapy, and surgical-received all play a role in making a prediction of MpBC.

Although existing studies have shown that patients with MpBC have a lower rate of regional node metastasis by around 22–31% (8, 31, 32), the prognosis of MpBC is affected by regional node involvement, as our results exhibited. Similar outcomes to those were concluded by other researchers (28). In a study of 90 patients with MpBC in China, Zhang et al. confirmed that regional node status was an independent predictor for OS (28). Lee et al. also came to the same conclusion that positive regional nodes would lead to a dismal clinical ending (33). In our study, mastectomy is another factor that worsens the prognosis of patients with MpBC. Previously published studies also ascertained profitable prognostic implications of BCS; on the contrary, no surgery and mastectomy play an opposite role (34). Some scholars, however, pointed out that the type of surgery was not a prognostic factor for disease-specific survival (DSS) or OS (35, 36). For a retrospective study, this discrepancy could be attributable to the likelihood of selection bias. Furthermore, our findings also stated whether or not receiving chemotherapy also affects the prognosis of MpBC. Conventional anthracycline combined with cyclophosphamide chemotherapy is ineffective and cannot benefit patients with MpBC (37). Even with the most effective paclitaxel-containing chemotherapy regimen, the clinical efficiency for patients with advanced MpBC is less than 20% (38). Thus, it can only be tentatively concluded that adjuvant chemotherapy containing paclitaxel is the most likely chemotherapy regimen to benefit MpBC patients (38). Despite the fact that there are still few viable treatment options for MpBC in the systemic setting, and the chemotherapy regimen as well as its efficacy are debatable, chemotherapy improves the prognosis of patients with MpBC (39). The involvement of chemotherapy in the prognosis of patients with MpBC has been described before (19). Chemotherapy was related to prolonged survival in the report of Rakha et al., albeit this impact was limited to early-stage disease (19). In addition, tumor stage is also related to the prognosis of MpBC. Tumor size adversely affects the OS of patients with MpBC. Several studies have found that the higher the tumor stage, the worse the prognosis of patients with MpBC, which matches our findings (3, 34, 40). Furthermore, the relationship between histological subtypes and MpBC prognosis was also discussed in many articles. Yamaguchi et al. disclosed significantly higher metastatic risks in MpBC containing spindle cells (41). MpBC rich in spindle cells manifests a more aggressive biobehavior (33). Intriguingly, other articles focused in depth and presented that MpBC with spindle cell appears with a higher frequency of PIK3CA mutation, which may benefit from radio-/chemotherapy (29).

In this study, 998 postoperative patients diagnosed with TN MpBC were extracted from the SEER database. After analysis, our findings imply that age, marital status, regional node metastasis, chemotherapy, and surgical type are all variables that influence OS; therefore, we developed a prediction model and a nomogram based on these variables. The predictive model can predict the 3-, 5-, and 8-year OS of patients with MpBC accurately and effectively, providing a valid scientific basis for predicting the prognosis of MpBC. Identification of these characteristics and an understanding of their role in disease aggressiveness and progression could lead to more customized treatment for this patient group. Further, it may contribute to the current knowledge on the MpBC management strategy.

Limitations do exist in our study. First, we acquired data from the SEER database, which lacked numerous valuable characteristics, including comorbidity, the specific chemotherapy regimen, radiotherapy dose, target volume, endocrine therapy, and pathological condition. Second, almost all the people we included were from Europe and America, and the Asian population needs to be further investigated. Third, this research was a retrospective analysis with weak argumentation.

### Conclusions

Using six clinicopathological elements, we developed a prediction model and nomogram for predicting the OS of postoperative patients with MpBC. The validation of the model has proven to be extremely effective. These methods can assist physicians with patient counseling and treatment decision-making in prognostic evaluation and tailored therapy, notwithstanding the need for further external validation.

## Data Availability Statement

The raw data supporting the conclusions of this article will be made available by the authors, without undue reservation.

## Ethics Statement

Ethical review and approval was not required for the study on human participants in accordance with the local legislation and institutional requirements. The patients/participants provided their written informed consent to participate in this study.

## Author Contributions

KZ analyzed and interpreted the patient data regarding the MpBC and was a major contributor in writing the manuscript. YC designed this study and performed most statistics analysis. RG provided initial research idea for this study. LD contributed to the editorial of tables and figures in this manuscript. JW, YT, and SZ provided practical suggestions for this research. DC and SH guided the development and conduct of the study. All authors listed have made a substantial, direct, and intellectual contribution to thework and approved it for publication.

## Funding

This work was supported by the National Natural Scientific Foundation of China (81860465 to DC and 82160532 to SH) and the Yunnan Applied Basic Research Projects-Union Foundation (202001AY070001-237 to SH).

## Conflict of Interest

The authors declare that the research was conducted in the absence of any commercial or financial relationships that could be construed as a potential conflict of interest.

## Publisher’s Note

All claims expressed in this article are solely those of the authors and do not necessarily represent those of their affiliated organizations, or those of the publisher, the editors and the reviewers. Any product that may be evaluated in this article, or claim that may be made by its manufacturer, is not guaranteed or endorsed by the publisher.
